# Inflammation and Cognition: The Moderating Role of Neighborhood Disadvantage

**DOI:** 10.1093/geroni/igaf122.2905

**Published:** 2025-12-31

**Authors:** Hye Won Chai, Abigail Stephan, Vanessa Martinez, Alexa Allan, Christopher Engeland, Alyssa Gamaldo, Lesley Ross

**Affiliations:** Clemson University, Clemson, South Carolina, United States; Clemson University, Clemson, South Carolina, United States; Clemson University, Clemson, South Carolina, United States; The Pennsylvania State University, University Park, Pennsylvania, United States; The Pennsylvania State University, University Park, Pennsylvania, United States; Clemson University, Clemson, South Carolina, United States; Clemson University, Clemson, South Carolina, United States

## Abstract

Extant literature suggests neighborhood disadvantage and inflammation as significant correlates of cognitive function among older adults. However, the joint effects of the contextual and biological factors on cognition have received less attention. The biopsychosocial model of dementia posits that understanding the interplay between biological and socio-contextual determinants of cognition is integral for more effective identification and prevention of Alzheimer’s disease and related dementias (ADRD). Therefore, this study examined whether and how the associations between inflammation and cognition vary by neighborhood disadvantage. Participants were drawn from a subsample of the Elucidating the Necessary Active Components of Training (ENACT, NCT05366023) study that assessed a wide range of biological, psychological, and social factors related to cognitive health from older adults aged 55 to 80 years (N = 51; Mean age=64.3). Inflammation was measured using C-reactive protein (CRP) and cognition was measured using Test My Brain (TMB) tasks. Neighborhood disadvantage was measured using the Area Deprivation Index (ADI). Results showed significant interactions between CRP and ADI for TMB tasks measuring processing speed (Digit Symbol Coding; accuracy) and working memory (Trail Making B; time to completion in milliseconds). Specifically, higher inflammation was associated with worse processing speed (B = -0.01; p < .05) and working memory (B = 8323.66; p < .1) only among those from more disadvantaged neighborhoods. Findings suggest older adults from socioeconomic disadvantaged neighborhoods may be more vulnerable to the cognitive health costs of inflammation, highlighting the need for tailored prevention and intervention efforts that specifically target older adults living in these neighborhoods.

